# Formation of Solid Solutions and Physicochemical Properties of the High-Entropy Ln_1−x_Sr_x_(Co,Cr,Fe,Mn,Ni)O_3−δ_ (Ln = La, Pr, Nd, Sm or Gd) Perovskites

**DOI:** 10.3390/ma14185264

**Published:** 2021-09-13

**Authors:** Juliusz Dąbrowa, Klaudia Zielińska, Anna Stępień, Marek Zajusz, Margarita Nowakowska, Maciej Moździerz, Katarzyna Berent, Maria Szymczak, Konrad Świerczek

**Affiliations:** 1 Faculty of Materials Science and Ceramics, AGH University of Science and Technology, Al. Mickiewicza 30, 30-059 Krakow, Poland; zajuszm@agh.edu.pl (M.Z.); margarita@student.agh.edu.pl (M.N.); szymczak@student.agh.edu.pl (M.S.); 2 Faculty of Energy and Fuels, AGH University of Science and Technology, Al. Mickiewicza 30, 30-059 Krakow, Poland; olszewska@agh.edu.pl (A.S.); mozdzier@agh.edu.pl (M.M.); xi@agh.edu.pl (K.Ś.); 3 Academic Centre for Materials and Nanotechnology, AGH University of Science and Technology, Al. Mickiewicza 30, 30-059 Krakow, Poland; kberent@agh.edu.pl; 4 AGH Centre of Energy, AGH University of Science and Technology, ul. Czarnowiejska 36, 30-054 Krakow, Poland

**Keywords:** high entropy oxides, perovskites, structural properties, electrical properties

## Abstract

Phase composition, crystal structure, and selected physicochemical properties of the high entropy Ln(Co,Cr,Fe,Mn,Ni)O_3−δ_ (Ln = La, Pr, Gd, Nd, Sm) perovskites, as well as the possibility of Sr doping in Ln_1*−x*_Sr*_x_*(Co,Cr,Fe,Mn,Ni)O_3−δ_ series, are reported is this work. With the use of the Pechini method, all undoped compositions are successfully synthesized. The samples exhibit distorted, orthorhombic or rhombohedral crystal structure, and a linear correlation is observed between the ionic radius of Ln and the value of the quasi-cubic perovskite lattice constant. The oxides show moderate thermal expansion, with a lack of visible contribution from the chemical expansion effect. Temperature-dependent values of the total electrical conductivity are reported, and the observed behavior appears distinctive from that of non-high entropy transition metal-based perovskites, beyond the expectations based on the rule-of-mixtures. In terms of formation of solid solutions in Sr-doped Ln_1−*x*_Sr*_x_*(Co,Cr,Fe,Mn,Ni)O_3−δ_ materials, the results indicate a strong influence of the Ln radius, and while for La-based series the Sr solubility limit is at the level of x_max_ = 0.3, for the smaller Pr it is equal to just 0.1. In the case of Nd-, Sm- and Gd-based materials, even for the x_Sr_ = 0.1, the formation of secondary phases is observed on the SEM + EDS images.

## 1. Introduction

The development of high-entropy materials is widely considered to be a major breakthrough in the design of next-generation functional compounds, with the high-entropy oxides (HEOx) having special prominence in this regard. Since their initial development in 2015, when Rost et al. synthesized a single-phase, rocksalt-structured (Co,Cu,Mg,Ni,Zn)O solid solution [1], the high-entropy design principle has been successfully translated to a number of different crystallographic structures, such as transition metal-based high-entropy spinels [2,3,4,5], high-entropy perovskites [6,7], bixbyite- and fluorite-structured high-entropy oxides [8,9,10,11], high-entropy lanthanide sesquioxides [12], magnetoplumbite-structured high-entropy oxides [13], high-entropy pyrochlores [14], and high-entropy garnets [15]. By analogy to the conventional oxide materials, among all those listed above, one group in particular attracts much attention of the scientific community, namely, the high-entropy perovskites. The classical ABO_3_ perovskites, where A and B denotes separate cation sublatticies, are widely considered to be among the most versatile functional materials, offering (through a proper selection of the chemical composition) possibility to tune the properties, yielding unmatched catalytic activity, excellent electrochemical performance, as well as unique optical, transport, magnetic, and other functional properties [16,17,18]. Consequently, they have found their way into numerous applications in catalysis, electronics, and energy conversion-related technologies [16,17,18,19].

As of today, by far the most studied perovskite systems are based on LnTMO_3_-type configuration, in which Ln denotes lanthanide (at the A-site), while TM denotes transition metal (at the B-site). At the same time, the wide range of substitution in all sublattices enables to obtain virtually unlimited number of different compositions. It is not surprising that a similar concept, i.e., usage of transition metals, became a basis to study the high-entropy regions of the phase diagrams of multicomponent ABO_3_-type oxides. The first report concerning such materials was published in 2018 by Sarkar et al. [6], who considered the application of the high-entropy approach to modify both cationic sublattices, including simultaneous substitutions at both, A and B sublattices. The A-site ions were selected from the group of Gd, La, Nd, Sm, and Y elements, while the B-site ones were chosen from the following TMs: Co, Cr, Fe, Mn, and Ni. A systematic pattern was adapted for the studied compositions: (5A_0.2_)CoO_3−δ_, (5A_0.2_)CrO_3−δ_, etc., and Gd(5B_0.2_)O_3−δ_, La(5B_0.2_)O_3−δ_, etc., where 5A_0.2_ and 5B_0.2_ denote equimolar combinations of all the listed A- and B-site cations, (Gd,La,Nd,Sm,Y) and (Co,Cr,Fe,Mn,Ni), respectively. Out of 11 proposed compositions, six proved to be single-phase perovskites crystallizing in orthorhombic *Pbnm* symmetry: (5A_0.2_)CoO_3−δ_, (5A_0.2_)FeO_3−δ_, Gd(5B_0.2_)O_3−δ_, La(5B_0.2_)O_3−δ_, Nd(5B_0.2_)O_3−δ_, and the complex (5A_0.2_)(5B_0.2_)O_3_ ((Gd,La,Nd,Sm,Y)(Co,Cr,Fe,Mn,Ni)O_3−δ_), comprising ten different cations in equimolar proportions. Interestingly, in the case of multiphase (5A_0.2_)MnO_3−δ_, the presence of the reversible phase transformation to the single-phase structure was observed upon heating, indicating that the configurational entropy of the system might actually prominently contribute to the stabilization of the solid solution. The Ln(5B_0.2_)O_3−δ_-type compositions were further studied in the follow-up paper with the main emphasis put on their magnetic properties [20]. The value of the Goldschmidt tolerance factor *t* was found to strongly correlate with the magnetic ordering temperature, showing the possibility of tailoring the properties of HEOx by careful selection of the composing ions. This effect is especially interesting in the light of data reported in the recent, very extensive review of the magnetic properties of HEOx, also published by the group of Sarkar et al. [21]. Based on the available literature, they were able to identify the common, unique features of the magnetic HEOx, distinguishing them from their conventional counterparts:

- long range antiferromagnetic (AFM) order is present despite the extreme ionic disorder and the high degree of dilution with non-magnetic ions;
- gradual magnetic transitions are spaced over a wide range of temperatures;
- short-range magnetic correlation is present well above Néel temperature (TN);
- Based on such characteristics it can be anticipated that the further development of perovskite-structured HEOx might result in obtaining of a number of potentially unique magnetic materials.

Most recently, however, the interest in the B-site disordered LnTMO_3_ high-entropy perovskites based on the Co, Cr, Fe, Mn, and Ni elements, has shifted toward their electrochemical performance. In the first study, Dąbrowa et al. [22] carried out an initial assessment of the possibility of utilizing the high-entropy perovskites as air-electrode materials for solid oxide fuel cell technology (SOFCs) on the example of La_1−*x*_Sr*_x_*(Co,Cr,Fe,Mn,Ni)O_3−δ_ perovskite. The introduction of Sr allowed the transport properties of the studied perovskites to be significantly enhanced, by means of both bandwidth and filling control mechanisms [23]. Based on characterization of the physiochemical properties, the La_0.7_Sr_0.3_(Co,Cr,Fe,Mn,Ni)O_3−δ_ composition (L7S3TM) was selected for examination as the potential air-electrode material. The studies proved its high inertness towards the La_0.8_Sr_0.2_Mg_0.2_Ga_0.8_O_3−δ_ (LSGM) solid electrolyte, with moderate thermal expansion values up to 1000 °C, and showed promising electrochemical performance in the fuel cell. Most importantly, the L7S3TM represents a rare case of Cr-containing air electrode material, which may contribute to its higher resistance to the deleterious Cr-poisoning effect, problematic for the state-of-the-art SOFCs [24]. The next study focused on the application of the La(Co,Cr,Fe,Mn,Ni)O_3−δ_ material as a potential catalyst for the oxygen evolution reaction (OER), essential for the water-splitting process [25]. The optimized, non-equimolar La(2Co,Cr,Fe,Mn,Ni)O_3−δ_ composition exhibited excellent performance, achieving OER overpotential of 325 mV at a current density of 10 mAcm^−2^, greatly outperforming not only all conventional LaTMO_3_ perovskites (TM = Co, Cr, Fe, Mn or Ni), but also state-of-the-art RuO_2_ catalyst. What is more, the material displayed excellent stability, with no sign of performance degradation after 50 h of testing. Such increased stability appears to be an inherent property of HEOx in nearly all electrochemical applications [26,27,28], making them even more promising in this context.

To maximize the electrochemical performance of the high-entropy perovskites, one can benefit from the most common mechanism utilized in the conventional perovskites, namely, the A-site doping with +2 alkaline-earth ions, such as Sr, Ba, and Ca [23]. This allows for both, control over the angle between neighboring TMO_6_ octahedra, which governs the effectiveness of the 3*d*_TM_-2*p*_O_ orbitals overlapping (bandwidth control [23]), and modification of the charge of B-site ions (including their multivalency), thanks to the presence of the (respective) charge compensation mechanism (filling control) [23]. The possibility of such modifications of the LnTMO_3_ high-entropy perovskites has already been successfully proven in [22]. Still, the selection of the A-site lanthanide is expected to play an equally important role concerning the overall properties of the system, directly influencing electronic and ionic conductivity [19], thermomechanical and magnetic properties [20,29], but also indirectly affecting the system through different available alkaline-earth dopant solubility limit [30]. The present study aims to evaluate the lanthanide selection on the structure and properties of base Ln(Co,Cr,Fe,Mn,Ni)O_3−δ_ perovskites, as well as on the influence of Ln on the solubility of the most typical A-site dopant-Sr.

## 2. Materials and Methods

The base Ln_1−x_Sr_x_(Co,Cr,Fe,Mn,Ni)O_3−δ_ (Ln = La, Pr, Nd, Sm or Gd) oxide powders were synthesized with the modified sol-gel method. The synthesis was carried out using the wet chemistry route proposed by Pechini [31]. In general, compounds synthesized by this method are characterized by highly homogeneous morphology and nano-sized grains, contributing to the method’s capability to obtain single-phase multi-component materials. As starting chemicals, the following nitrates, provided by the Alfa Aesar company, were used: La(NO_3_)_3_·6H_2_O (99.9%), Pr(NO_3_)_3_·6H_2_O (99.99%), Nd(NO_3_)_3_·6H_2_O (99.9%), Sm(NO_3_)_3_·6H_2_O (99.9%), Gd(NO_3_)_3_·6H_2_O (99.9%), Sr(NO_3_)_2_ (99.97%), Co(NO_3_)_2_·6H_2_O (98.0–102.0%), Cr(NO_3_)_3_·9H_2_O (98.5%), Fe(NO_3_)_3_·9H_2_O (98.0–101.0%), Mn(NO_3_)_2_·6H_2_O (98.0%) and Ni(NO_3_)_2_·6H_2_O (99.99%). Citric acid monohydrate (Alfa Aesar 99.5+%) was added in the amount equal to two times the molar sum of cations, followed by the addition of ethylene glycol in the molar amount of four times the sum of the cations. In a typical synthesis, the chemicals were dissolved in 200 mL of distilled water. The mixture was then placed on the magnetic stirrer set at 300 rpm. Initially, temperature was set at 150 °C. After 15 min, it was increased to 300 °C and kept until the evaporation of water was completed. The precursors were calcined at 700 °C for 6 h in air, followed by slow cooling with a furnace. All the powders obtained were formed into pellets of 10 mm diameter, using a uniaxial hydraulic press with a vacuum pump, with a subsequently applied pressure of 0.5, 1, and 1.5 tones. The pellets were then sintered in a tube furnace for 20 h at 1000 °C and then quenched to room temperature (RT). For the dilatometry studies, the pellets were prepared at 1200 °C, to allow for a higher density of the sinters. 

The morphology of the obtained samples was examined using the scanning electron microscopy method (SEM, FEI Versa 3D, 15 kV accelerating voltage). Homogeneity and chemical composition were examined by the energy-dispersive X-ray spectroscopy (EDS, Oxford Instruments Ultim Max). 

X-ray diffraction (XRD, Panalytical Empyrean diffractometer with Cu Kα radiation) measurements at RT were performed using the Bragg–Brentano geometry, within the 10–90 deg angular range. The obtained data were analyzed with the use of Malvern Panalytical X’Pert HighScore Plus 2.0 software (Malvern, UK) and ICDD PDF2 database. Rietveld analysis was performed in order to determine the respective content of phases, unit cell parameters, and theoretical density.

Measurements of thermal expansion coefficient (TEC, Linseis L75 Platinum Series dilatometer) were also performed. Samples were heated to 900 °C at a rate of 5 °C/min and then cooled to RT in the same conditions. Dense, cuboid-shaped samples with a length of up to 8 mm were placed between alundum discs pressing the sample with a force of 300 N. The correction curve resulting from the expansion of the alumina discs and holder was subtracted from the recorded values.

Total electrical conductivity was studied with electrochemical impedance spectroscopy (EIS, Zurich Instruments MFIA 5MHz analyzer with quasi-4 probe configuration). The sintered pellets were prepared for measurements by applying platinum paste on the respective surfaces. Subsequently, samples were dried at 90–100 °C and fired at 1000 °C in air. The measurements were carried out in air. Due to the lack of capacitance-related contribution, a simple RL equivalent circuit was used for the analysis. The temperature range was 50–900 °C, with 50 °C steps. The measurements were performed during the cooling run.

In order to evaluate oxygen content changes with temperature of selected materials, thermogravimetric (TG, TA Q5000 IR thermobalance) method was utilized, with measurements (two consecutive cycles) performed in synthetic air in the RT to 850 °C temperature range, with 5 °C min^−1^ heating and cooling rates.

## 3. Results and Discussion

### 3.1. Crystal Structure of Ln(Co,Cr,Fe,Mn,Ni)O_3−δ_

**Figure 1:**
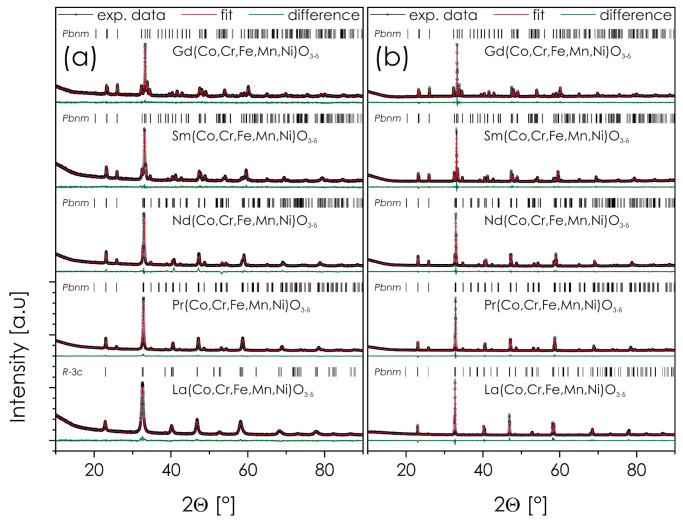
X-ray diffraction (XRD) diffractograms of the Ln(Co,Cr,Fe,Mn,Ni)O_3−δ_ (Ln = La, Pr, Gd, Nd, Sm) materials: (**a**) as-calcined powders; (**b**) pellets quenched after sintering at 1000 °C for 20 h.

X-ray diffraction data for all base Ln(Co,Cr,Fe,Mn,Ni)O_3−δ_ compositions, i.e., without strontium doping, are presented in Figure 1 together with Rietveld refinements, for both, as-calcined powders and quenched pellets. 

The systematic structural data obtained with the respective refinement residuals are gathered in Table 1. The data for La(Co,Cr,Fe,Mn,Ni)O_3−δ_ come from our previous study [22].

As can be seen, all compositions turned out to be single-phase, which somewhat differs from the results of Sarkar et al., who reported the presence of secondary phases for the as-received Sm(Co,Cr,Fe,Mn,Ni)O_3−δ_ [6]. This shows the benefits of the application of the Pechini method, when compared with the nebulized spray pyrolysis (NSP), utilized by the cited authors. Composition with praseodymium, which has never been investigated before, displays similar structural properties to other studied materials in the series. For the as-calcined powders, all compositions exhibit orthorhombic perovskite structure with the *Pbnm* space group, except for the La-based material, in which case the higher symmetry *R*-3*c* space group gave better fitting results. This is likely due to the largest ionic radius for this element among considered ones, resulting in the closer-to-ideal value of Goldschmidt’s tolerance factor [32]. Importantly, calculated values of the ratio between the lattice parameters *a*, *b*, and *c* shows that the distortion of the structure generally increases with the decreasing ionic radius of the Ln ion (Table 1). 

All sintered and quenched samples are characterized by the presence of a lower-symmetry *Pbnm* space group. In the case of La-based material, this is also accompanied by smaller unit cell volume, as well as significantly narrowed X-ray reflections. It seems, therefore, that after annealing at 1000 °C crystallinity of the material improved, and total oxygen content changed (likely increased, resulting in the unit cell shrinkage), which is further supported by the results of TG measurements, see Figure S1. All that contributed to the lowering of the crystal’s symmetry.

**Figure 2:**
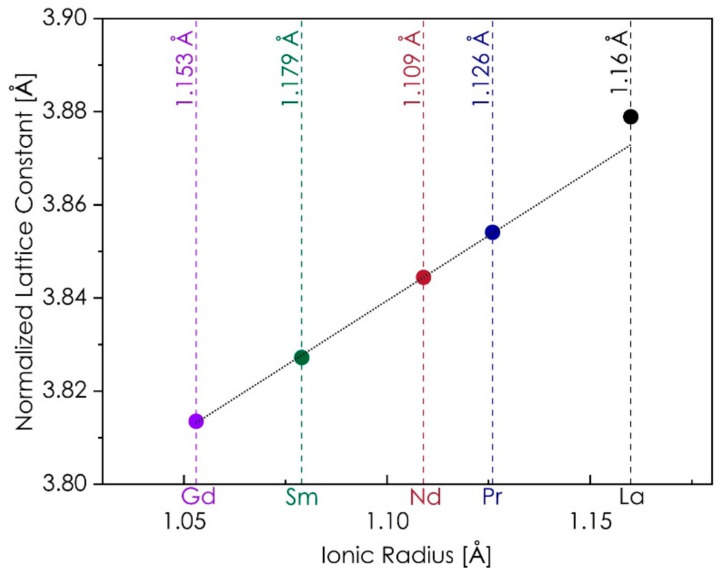
Relation between the quasi-cubic normalized lattice constant *a*_0_ of Ln(Co,Cr,Fe,Mn,Ni)O_3−δ_ and ionic radii of the respective Ln ions (for eight-fold coordination [34]). Data for the pellets quenched after sintering at 1000 °C for 20 h. The errors are not marked, as they are within the size of data points. The linear fit for Gd, Sm, Nd, and Pr is extrapolated on the ionic radius of La.

The influence of the lanthanide ion type on the lattice parameters is easily seen for the normalized, quasi-cubic parameters, also provided in Table 1. The normalized cell volume *V*_0_ and quasi-cubic normalized lattice constant *a*_0_ were calculated assuming that the equivalent cubic unit cell is 4-times smaller than the orthorhombic one (2*a*_0_ × √2*a*_0_ × √2*a*_0_), and the rhombohedral is 6-times smaller [33]. Relation between the normalized lattice constant *a*_0_ and the ionic size of the A-site cations [34] is presented in Figure 2. Due to the lack of complete data for ionic radii of selected lanthanides in 12-fold coordination, the 8-fold values were used, which in fact better represent the actual A-site coordination in a highly distorted, *Pbnm* perovskite structure [33]. As expected, the normalized lattice constant increases monotonically with the increase in ionic radius of the A-site rare-earth element. Interestingly, the lattice constant for La-based composition is slightly bigger than the value based on linear extrapolation. This can be explained by the tendency of lanthanum to be placed in surrounding with effectively higher coordination number, in which it exhibit larger radius [34].

### 3.2. Scanning Electron Microscopy Plus Energy-Dispersive X-ray Spectroscopy (SEM+EDS) Analysis of the Ln(Co,Cr,Fe,Mn,Ni)O_3−δ_ Series

**Figure 3:**
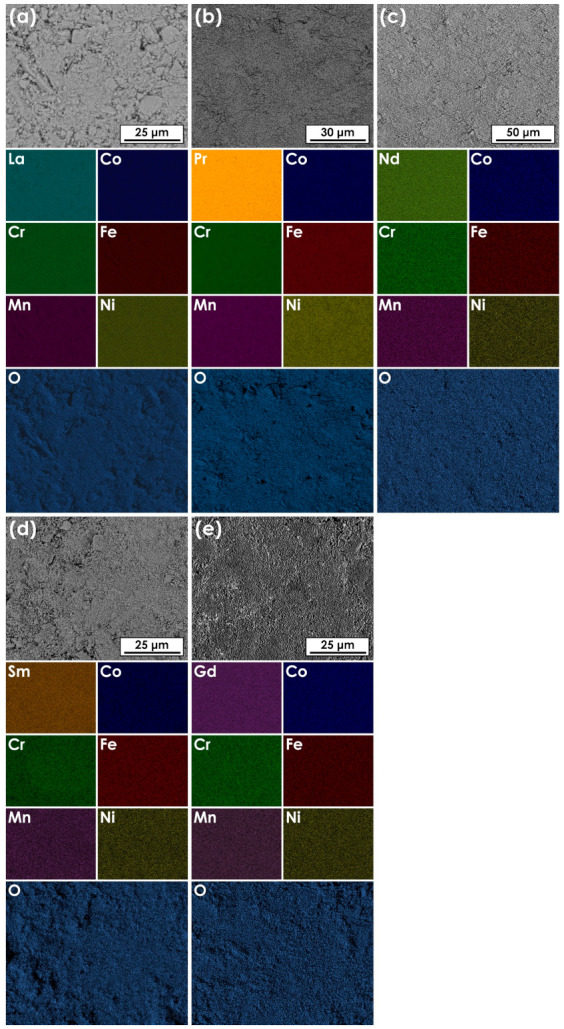
Results of the EDS mappings for pellets sintered at 1000 °C for 20 h followed by quenching: (**a**) La(Co,Cr,Fe,Mn,Ni)O_3−δ_; (**b**) Pr(Co,Cr,Fe,Mn,Ni)O_3−δ_; (**c**) Nd(Co,Cr,Fe,Mn,Ni)O_3−δ_; (**d**) Sm(Co,Cr,Fe,Mn,Ni)O_3−δ_; e) Gd(Co,Cr,Fe,Mn,Ni)O_3−δ_.

The sintered pellets of all compositions were examined with the use of SEM and EDS methods to assess their morphology and homogeneity. In the case of La-based material, the data were supplemented from our previous study, and can be taken as a reference [22]. The results of EDS mappings for all materials are presented in Figure 3, while the evaluated average chemical composition, measured by the EDS area analysis, are summarized in Table 2. 

As is visible, thanks to the application of wet chemistry route, it was possible to obtain homogeneous materials with relatively small grain size. Considering previously discussed XRD results, it can therefore be stated that all base Ln(Co,Cr,Fe,Mn,Ni)O_3−δ_ compositions are single-phase and exhibit a high degree of chemical composition homogeneity, with no segregation of any element taking place. Also, relative ratios of the TM elements remain close to the assumed ones for all compositions.

### 3.3. Thermomechanical Behavior of the Ln(Co,Cr,Fe,Mn,Ni)O_3−δ_ Series

**Figure 4:**
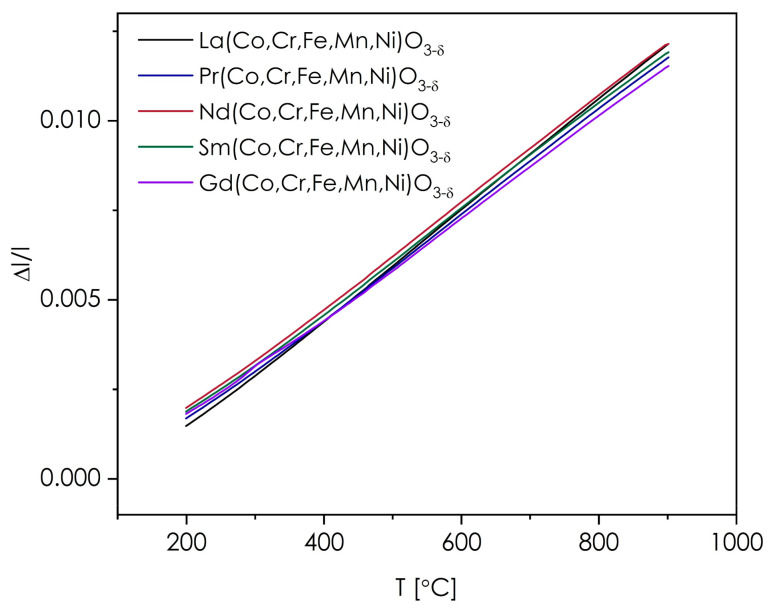
Results of the dilatometric measurements of the Ln(Co,Cr,Fe,Mn,Ni)O_3−δ_ sinters (heating run).

In order to examine the thermal expansion coefficient (TEC), all base materials were subjected to the dilatometeric measurements. As mentioned above, in this case, the sintering temperature of 1200 °C was used to further densify the samples. The results (gathered on the heating run) are presented in Figure 4, while the determined values of TEC are summarized in Table 3.

The thermomechanical behavior within the Ln(Co,Cr,Fe,Mn,Ni)O_3−δ_ series is similar for all compositions, although the correlation between the ionic radius of lanthanide ion and the value of TEC occurs—in general, the smaller the radius, the lower is the thermal expansion coefficient, which is especially evident for the La-based composition (Table 3). Such behavior is in line with that typically reported for the conventional LnTMO_3_ perovskites [29], as well as other perovskite-type structures [35]. No sign of chemical expansion due to the oxygen release from the structure (i.e., increase of TEC) can be observed at higher temperatures, indicating that the materials maintain their total oxygen content up to 900 °C without any significant decrease. This appears as advantageous, since chemical expansion is a major problem for many state-of-the-art perovskites (especially Co-containing ones), which are used for manufacturing air electrodes for SOFCs [22]. The TEC values can be considered as moderate, differing from those of the binary LaTMO_3_ perovskites: LaFeO_3_ (ca. 11.6 × 10^−6^ K^−1^ [36]), LaCrO_3_ (9.5 × 10^−6^ K^−1^ [29]), LaMnO_3_ (11.2 × 10^−6^ K^−1^ [29]) and LaCoO_3_ (23.5 × 10^−6^ K^−1^ [29]), and being lower than those reported e.g., for the LSCF series (La_1−*x*_Sr*_x_*Co_1−*y*_Fe*_y_*O_3−*δ*_) [37]. Importantly, the results are matching well thermal expansion of typical solid electrolytes used in SOFC technology, e.g., Ce_0.8_Gd_0.2_O_1.9_ (12.4 × 10^−6^ K^−1^ [38]) and La_0.8_Sr_0.2_Ga_0.8_Mg_0.2_O_2.85_ (11.1 × 10^−6^ K^−1^ [39]). Notably, in the case of La(Co,Cr,Fe,Mn,Ni)O_3−δ_ the determined TEC is slightly lower than that reported for La_0.7_Sr_0.3_(Co,Cr,Fe,Mn,Ni)O_3−δ_ (16.0(3) × 10^−6^ K^−1^) [22], which is consistent with the expectations based on the behavior of conventional perovskite systems [29].

### 3.4. Electrical Conductivity of the Ln(Co,Cr,Fe,Mn,Ni)O_3−δ_ Series

**Figure 5:**
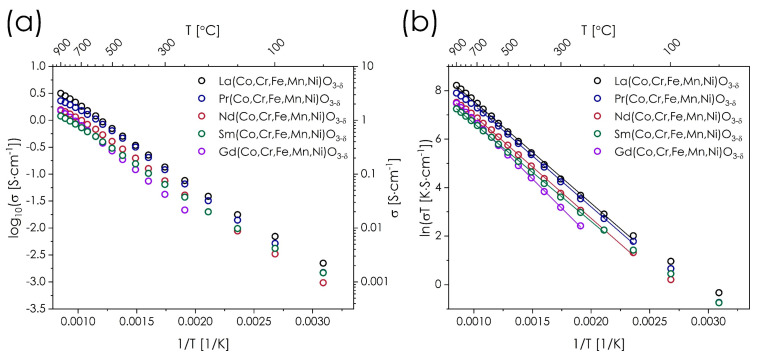
(**a**) Value of the total electrical conductivity of the Ln(Co,Cr,Fe,Mn,Ni)O_3−δ_ series; (**b**) total electrical conductivity of the Ln(Co,Cr,Fe,Mn,Ni)O_3−δ_ series presented in Arrhenius coordinates. The linear fits used for the determination of energy of activation are also presented.

The value of the electrical conductivity is one of the essential properties for functional materials, especially with respect to the energy conversion technologies. Measured total electrical conductivity for the Ln(Co,Cr,Fe,Mn,Ni)O_3−δ_ series as a function of temperature is presented in Figure 5a. In the case of the Gd(Co,Cr,Fe,Mn,Ni)O_3−δ_ composition, only the range of 250–900 °C is presented, due to experimental errors connected with its lower conductivity in the low-temperature range.

As can be seen in Figure 5a, all materials exhibit very similar, thermally activated, semiconducting-type temperature dependence of the total electrical conductivity within the whole measured temperature range. However, practically in all cases, a small decrease of the slope value can be observed at high temperatures, indicating that the mechanism of electronic conduction involves polaron hopping [37], but alternatively, such behavior may also stem from a limited oxygen release from the structure, which causes disruption of TM-O-TM conduction pathways [37]. To provide a deeper insight, the conductivity data were also presented in the ln(*σT*) = *f*(1/*T*)-type coordinates (Figure 5b). Much better linearity of plots was achieved in this case, suggesting the adiabatic small polaron hopping mechanism, which is consistent with the behavior reported for binary LaFeO_3_, LaMnO_3_, and LaCrO_3_ perovskites [37,40]. Nevertheless, data for Pr- and Nd-containing materials at highest temperatures still indicate a small deviation from linearity, which likely stems from a fact that only these two lanthanides (among studied ones) can be present in the mixed-valence state [41,42]. Interestingly, neither of these materials appear to release oxygen at high temperatures in a rate different than e.g., La-based perovskite, see Figure S1. More studies are needed, however, to confirm (or disprove) the possible presence and influence of Nd and Pr mixed-valence state.

The summary regarding the values of conductivity, as well as determined energies of activation can be found in Table 4.

The values from Table 4 clearly show the similarities between all compositions from the studied Ln(Co,Cr,Fe,Mn,Ni)O_3−δ_ series, with the electrical conductivity (generally) increasing with the increase of lanthanide ionic radius. Also, the results can be correlated with the mean TM-O distance [43,44] generally decreasing with increasing value of σ_max_, see Figure S2. This is likely due to the decrease of structural distortion and better overlapping of the 3*d_TM_*-2*p_O_* orbitals [23]. The only exception is Gd(Co,Cr,Fe,Mn,Ni)O_3−δ_, which due to its higher activation energy, conducts better than Sm(Co,Cr,Fe,Mn,Ni)O_3−δ_ at high temperatures. Nevertheless, the obtained values vary at 900 °C only in a small range between 1.2 and 3.2 S·cm^−1^, showing that the occurring differences in transport properties are relatively small. 

Particularly interesting observations can be made when comparing the results for the La(Co,Cr,Fe,Mn,Ni)O_3−δ_ with data for a very similar La(Co,Cu,Fe,Mn,Ni)O_3−δ_ composition, studied by Han et al. [45], for which a distinctively different behavior was reported, both in terms of maximum conductivity value and the general character of the thermal dependence. Despite replacing just one of the ions, namely Cu for Cr, the reported *σ_max_* was nearly two orders of magnitude higher, exceeding 171 S·cm^−1^, with a much lower energy of activation. Such behavior is rather surprising, taking into account the conductivities of some of the binary LaTMO_3_ perovskites: LaFeO_3_ (ca. 0.8 S·cm^−1^ at 1000 °C [46]), LaCrO_3_ (ca. 1 S·cm^−1^ at 1000 °C [29]), LaMnO_3_ (ca. 115 S·cm^−1^ at 950 °C [29]), and LaCoO_3_ (ca. 1025 S·cm^−1^ at 700 °C [29]). If one considers Cr as the main element detrimental to the total conductivity value (as usually the combination of Co and Fe allows for much better conductivities [37,47], than those of studied HEOx), its addition at the level of 20% at the B-site positions should not result in deterioration of conductivity to such low values as presented in Figure 5a. This assumption may be supported by the studies of Cr influence on La(Cr,Mn)O_3_ system presented by Raffaelle et al. [40]. Furthermore, occupation of 20% of all the B-sites is below the accepted percolation threshold (~30% [48]), and therefore it is not probable that Cr ions can block three-dimensional charge transfer along TM–O–TM. This, combined with relatively high values of the activation energy of conduction, if compared with the binary perovskites and the mentioned La(Co,Cu,Fe,Mn,Ni)O_3−δ_ (i.e., generally not exceeding 0.2 eV), suggests that the observed behavior falls outside of the one anticipated by the rule-of-mixtures. This shows the prominent influence of synergistic effects related to the presence of this particular combination of B-sites ions in the studied materials. It may be speculated that the deleterious influence of Cr on the transport properties may result from the presence of such cations in highly-distorted (high entropy) B-site sublattice, in which their tendency to trap small polarons becomes unusually high, which is not observed in classical systems, although confirming it would require further, dedicated studies. Relating the discussed observations with the reported excellent catalytic activity of La(Co,Cr,Fe,Mn,Ni)O_3−δ_, exceeding that of the binary perovskites [25], shows a great potential of the high-entropy approach in generating unusual sets of physicochemical properties in (perovskite-type) materials.

### 3.5. Strontium Solubility in Ln_1−x_Sr_x_(Co,Cr,Fe,Mn,Ni)O_3−δ_ Series

As mentioned before, the A-site doping by alkaline-earth ions is by far the most popular solution of enhancing the transport and catalytic properties of the perovskite systems [22]. Due to the compatibility, in terms of ionic radius, with most of the lanthanide ions, strontium can be considered the most popular dopant in this regard (the ionic radius of Sr and La in 12-fold coordination is 1.44 and 1.36 Ǻ, respectively [34]) [22]. Therefore, from the point of view of potential applications of the reported Ln(Co,Cr,Fe,Mn,Ni)O_3−δ_ perovskites, it is crucial to determine Sr solubility limits for materials with different Ln, providing information on how far the properties of the parent compositions can be modified. 

**Figure 6:**
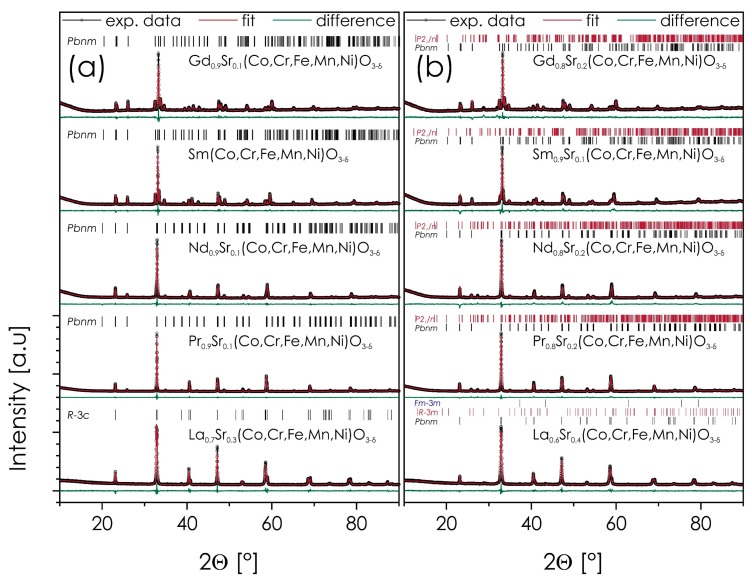
XRD diffractograms of the selected Ln_1−*x*_Sr*_x_*(Co,Cr,Fe,Mn,Ni)O_3−δ_ (Ln = La, Pr, Gd, Nd, Sm) pellets, sintered at 1000 °C for 20 h and quenched to RT: (**a**) data for compositions just below Sr solubility limit; (**b**) compositions with Sr content higher than the solubility limit.

In view of the above, the results of the X-ray diffraction experiments for selected, quenched sinters of the Ln_1−*x*_Sr*_x_*(Co,Cr,Fe,Mn,Ni)O_3−δ_ (Ln = La, Pr, Gd, Nd, Sm) materials are presented in Figure 6. Data for La-based series comes from our previous study [22]. The remaining diffractograms can be found in the Supplementary section, Figures S3–S6. Also, the results of the Rietveld refinements are presented in the supplement for the as-calcined powders and quenched pellets, respectively, together with the procedure’s residuals, see Tables S1 and S2. 

As can be seen, the behavior for both, as-calcined powders and quenched pellets, is not entirely consistent. In the case of the as-calcined powders, the Sr solubility limit for Ln_1−*x*_Sr*_x_*(Co,Cr,Fe,Mn,Ni)O_3−δ_ appears to be *x_max_* = 0.3, 0, 0.1, 0, and 0.1, for La, Pr, Nd, Sm, and Gd, respectively. Surprisingly, neither Pr- nor Sm-based series appear to be able to incorporate Sr into the structure and, as a result, no tendency that could be associated with the lanthanide ionic radius can be identified at this stage. In all cases, the secondary phase is SrCrO_4_, except for the La-based series, where peaks coming from Sr(CO_3_)_2_ are visible [22]. Furthermore, in the case of the La-based series the solubility limit is much higher than in the other series; also, the materials exhibit higher symmetry (*R*-3*c* vs. *Pbnm*), although precise interpretation is somewhat limited due to the nanosized grains of the analyzed powders, resulting in relatively broad peaks.

For Ln_1−*x*_Sr*_x_*(Co,Cr,Fe,Mn,Ni)O_3−δ_ sinters solubility limits (according to the XRD data) are as follows: *x_max_* = 0.3, 0.1, 0.1, 0, and 0.1, for La, Pr, Nd, Sm, and Gd, respectively. Also in this case, SrCrO_4_ constitutes the minority phase. However, in the case of the La-based series the main secondary phase is Sr_3_(CrO_4_)_2_, again proving the distinctively different behavior of these compositions, when compared with materials based on other lanthanides. It appears that after annealing the Pr-based perovskite is capable of incorporating low levels of strontium content, even though it is still significantly less than in the La-based series.

**Figure 7:**
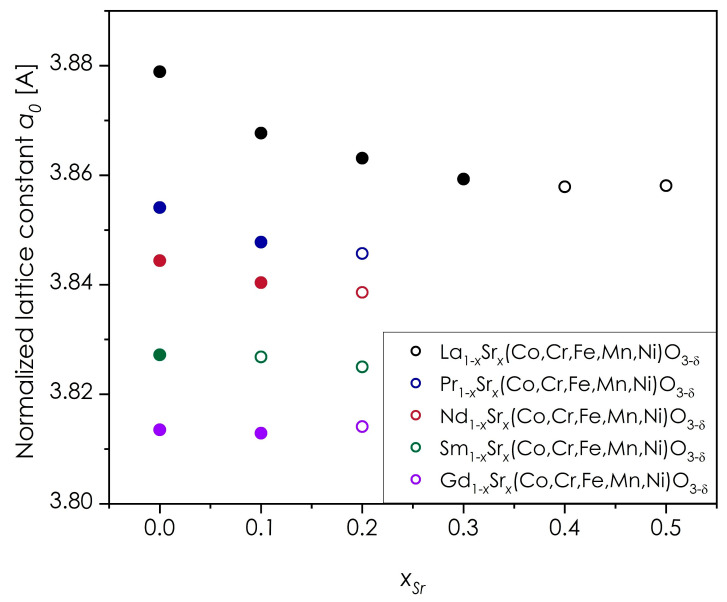
Normalized quasi-cubic lattice constant for the Ln_1−*x*_Sr*_x_*(Co,Cr,Fe,Mn,Ni)O_3−δ_ series. All data for pellets sintered at 1000 °C for 20 h, followed by quenching. Full circles mark compositions determined as single-phase by XRD, empty circles show values for the main perovskite phase in the multiphase compositions.

The dependence of the normalized quasi-cubic lattice constant as a function of the Sr content for each series is presented in Figure 7.

As is visible, in the case of La-based series, the a_0_ value is decreasing with the growing Sr content but becomes constant above *x* = 0.3, which corresponds to the previously identified solubility limit [22]. In fact, similar relation between strontium content and the value of normalized lattice parameter can be observed for all series, except only for the Gd-based material with the assumed highest level of Sr doping of 0.1. Such behavior across the whole Ln selection, indicates the presence of identical charge compensation mechanism for all of the doped, single-phase samples, likely originating from the oxidation of B-site transition metal ions, as can be derived from the electroneutrality principle with neglected oxygen content changes. For other than La-based series, this dependence is much less steep, which also supports their much lower solubility limits. It is also worth noting that for both, Sm- and Gd-based oxides, the normalized lattice constant is practically constant between *x_Sr_* equal to 0 and 0.1, suggesting that the Sr solubility limit is very restricted. Therefore, the Gd_0.9_Sr_0.1_(Co,Cr,Fe,Mn,Ni)O_3−δ_ apparently identified as single-phase may in fact be contaminated, but the secondary phase could not be identified due to the insufficient sensitivity of the XRD method. This effect is further discussed below. However in general, even though limited, the Sr solubility limit in Ln_1−*x*_Sr*_x_*(Co,Cr,Fe,Mn,Ni)O_3−δ_ series appears to increase with the growing size of lanthanide cation.

**Figure 8:**
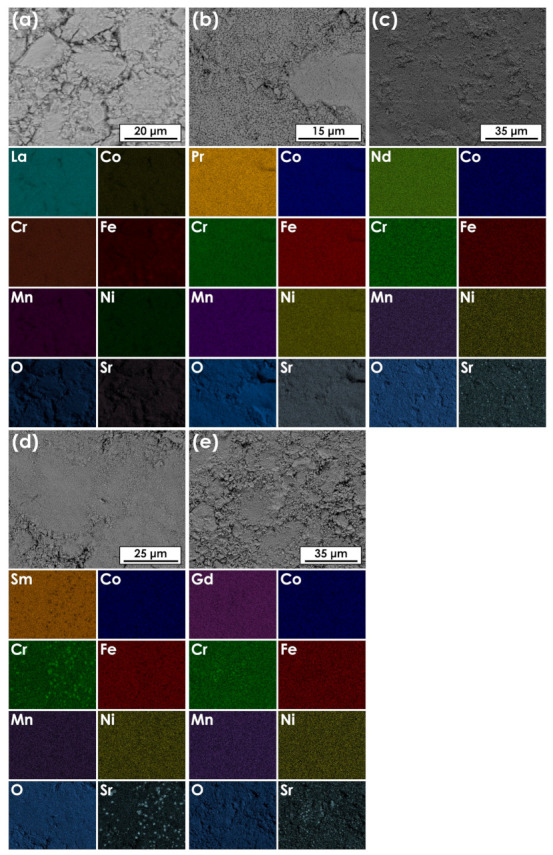
Results of the EDS mappings for pellets sintered at 1000 °C for 20 h followed by quenching. The compositions are within the solubility limit of Sr as established by XRD (except for Sm-based one with apparently no solubility): (**a**) La_0.9_Sr_0.1_(Co,Cr,Fe,Mn,Ni)O_3−δ_ [22]; (**b**) Pr_0.9_Sr_0.1_(Co,Cr,Fe,Mn,Ni)O_3−δ_; (**c**) Nd_0.9_Sr_0.1_(Co,Cr,Fe,Mn,Ni)O_3−δ_; (**d**) Sm_0.9_Sr_0.1_(Co,Cr,Fe,Mn,Ni)O_3−δ_; (**e**) Gd_0.9_Sr_0.1_(Co,Cr,Fe,Mn,Ni)O_3−δ_.

To supplement structural analysis, SEM + EDS studies were also performed for the considered samples. Exemplary results for materials with *x* = 0.1 are shown in Figure 8.

The comparison of the EDS mappings with the results of XRD measurements suggests even lower Sr solubility than anticipated. In fact, together with the La-based composition, only in the case of Pr_0.9_Sr_0.1_(Co,Cr,Fe,Mn,Ni)O_3−δ_ good homogeneity was observed (for Pr_0.8_Sr_0.2_(Co,Cr,Fe,Mn,Ni)O_3−δ_ see Figure S7). In all other cases, despite the lack of secondary phase-related signal on the X-ray diffractograms, Sr and Cr-enriched regions can be clearly seen. This agrees with the presence of SrCrO_4_ contamination, as well as indicates a more complex mechanism related to precipitation of the secondary phases. Importantly, point analyses show that the composition of the perovskite phase (excluding the precipitated Cr) is always close to the nominal one, also in the case of Sr content, despite exceeding *x_max_*. It can be stated, therefore, that Cr depletion actually results in the increased Sr solubility limit in the remaining perovskite phase. In the case of La- and Pr-based series, an interesting aspect connected with the Sr solubility might be investigation of the possible order-disorder transformations, as the composing TM ions may exhibit drastically different properties with regard to this phenomenon [49,50]. The potential influence of the high-entropy occupancy of the cation sublattice on the order–disorder transformation has been already reported by Witte et al. [20], who observed such transformation at 900 °C in the A-site disordered (Gd,La,Nd,Sm,Y)MnO_3−δ_. However, such studies in our materials would require additional, highly-detailed calorimetric and HT-XRD measurements, which are beyond the scope of the current paper.

## 4. Conclusions

The structure and properties of the Ln(Co,Cr,Fe,Mn,Ni)O_3−δ_ (Ln = La, Pr, Gd, Nd, Sm) materials are discussed in this work. The use of the Pechini method allowed for synthesizing all base compositions in the form of single-phase solid solutions, with the normalized, quasi-cubic lattice parameter increasing with the radius of the lanthanide ion. The thermal expansion is similar across the series, with apparently no chemical expansion, as well as a small increase of TEC values with the increasing size of the A-site ion. In the case of the electrical conductivity, all materials appear to exhibit an adiabatic small polaron hopping mechanism, with the maximum recorded conductivity values at 900 °C being surprisingly low, and varying between 1.2 and 3.2 S·cm^−1^. The comparison with the conventional perovskite systems indicates that the electrical behavior of Ln(Co,Cr,Fe,Mn,Ni)O_3−δ_ materials goes beyond the typical rule-of-mixtures, with both, high values of the energies of activation (0.35–0.42 eV) and lower-than-expected electrical conductivity. It suggests the presence of high entropy-related additional interactions between the B-site cations, affecting TM–O–TM charge transfer. To examine the possibility of modification of the functional properties of the studied materials, the solubility of Sr as the A-site dopant was examined. The results for the Ln_1−*x*_Sr*_x_* (Co,Cr,Fe,Mn,Ni)O_3−δ_ (Ln = La, Pr, Nd, Sm, Gd) series indicate a distinctively different behavior of the La-based materials, with the strontium solubility limit at the level of *x_max_* = 0.3, much higher than that for the next biggest cation, namely Pr, for which *x_max_* = 0.1. As for the oxides based on smaller lanthanides (Nd, Sm, Gd), none of the examined systems appear to be able to preserve the single-phase structure, even with the addition of *x_Sr_* = 0.1. However, the actual mechanism appears more complicated, with simultaneous precipitation of Cr-based oxides, as well as Sr incorporation into Cr-depleted perovskite phase.

## Figures and Tables

**Table 1:** The results of the Rietveld refinement with the procedure’s residuals for the Ln(Co,Cr,Fe,Mn,Ni)O_3−δ_ (Ln = La, Pr, Nd, Sm, Gd) series.

**As-Calcined Powders (Calcined at 700 °C for 6 h, Cooled Down with the Furnace)**											
	Phase	*ρ_theo_* (g/cm^3^)	*a* (Å)	*b* (Å)	*c* (Å)	*V* (Å^3^)	*a*_0_ (Å)	Rwp (%)	Gof	$b/\sqrt{2}a$	$c/\sqrt{2}a$
La(Co,Cr,Fe,Mn,Ni)O_3−δ_	*R*-3*c*	6.91	5.5086(2)	-	13.371(1)	58.563(6)	3.8834(1)	2.82	1.05	-	-
Pr(Co,Cr,Fe,Mn,Ni)O_3−δ_	*Pbnm*	7.10	7.6965(3)	5.4709(2)	5.4411(2)	57.277(4)	3.8547(1)	3.18	3.25	1.0053	0.9998
Nd(Co,Cr,Fe,Mn,Ni)O_3−δ_	*Pbnm*	7.26	7.6703(4)	5.4742(3)	5.4090(3)	56.779(6)	3.8435(1)	5.01	3.52	1.0093	0.9973
Sm(Co,Cr,Fe,Mn,Ni)O_3−δ_	*Pbnm*	7.53	7.6169(4)	5.5062(3)	5.3525(3)	56.121(5)	3.8266(1)	2.69	1.33	1.0223	0.9938
Gd(Co,Cr,Fe,Mn,Ni)O_3−δ_	*Pbnm*	7.81	7.5724(3)	5.5370(3)	5.2068(3)	55.522(4)	3.8149(1)	2.13	1.25	1.0341	0.9724
**Sinters (sintered at 1000 °C for 20 h, cooled down by quenching)**											
	Phase	*ρ_theo_* (g/cm^3^)	*a* (Å)	*b* (Å)	*c* (Å)	*V* (Å^3^)	*a*_0_ (Å)	Rwp (%)	Gof	$b/\sqrt{2}a$	$c/\sqrt{2}a$
La(Co,Cr,Fe,Mn,Ni)O_3−δ_	*Pbnm*	6.92	7.7472(2)	5.5116(1)	5.4671(1)	58.360(2)	3.8789(1)	7.17	2.01	1.0061	0.9980
Pr(Co,Cr,Fe,Mn,Ni)O_3−δ_	*Pbnm*	7.10	7.6976(1)	5.4673(1)	5.4413(1)	57.250(1)	3.8541(1)	3.77	5.52	1.0045	0.9997
Nd(Co,Cr,Fe,Mn,Ni)O_3−δ_	*Pbnm*	7.26	7.6728(1)	5.4751(1)	5.4099(1)	56.817(1)	3.8444(1)	4.06	2.60	1.0091	0.9971
Sm(Co,Cr,Fe,Mn,Ni) O_3−δ_	*Pbnm*	7.54	7.6178(1)	5.4987(1)	5.3532(1)	56.059(1)	3.8272(1)	3.05	1.96	1.0208	0.9938
Gd(Co,Cr,Fe,Mn,Ni) O_3−δ_	*Pbnm*	7.82	7.5726(1)	5.5293(1)	5.2980(1)	55.458(1)	3.8135(1)	2.53	1.89	1.0326	0.9894

**Table 2:** Average compositions of the Ln(Co,Cr,Fe,Mn,Ni)O_3−δ_ pellets sintered at 1000 °C for 20 h, followed by quenching, as determined by EDS area analysis (error of determination: 0.2–0.9 at.% for cations). The respective ratios for the B-site transition metal cations are also provided, under the assumption that the sum of their molar fractions is equal to 1.

**Average Composition (at.%)**							
	Ln	Co	Cr	Fe	Mn	Ni	O
La(Co,Cr,Fe,Mn,Ni)O_3−__δ_	22.9	4.6	6.0	4.8	4.3	4.4	53
Pr(Co,Cr,Fe,Mn,Ni)O_3−__δ_	24.1	4.6	4.5	4.7	4.9	4.8	53
Nd(Co,Cr,Fe,Mn,Ni)O_3−__δ_	25.6	4.6	5.1	5.4	5.2	4.7	49
Sm(Co,Cr,Fe,Mn,Ni)O_3−__δ_	25.8	5.1	5.0	5.6	5.2	4.4	49
Gd(Co,Cr,Fe,Mn,Ni)O_3−__δ_	23.5	5.7	4.8	5.1	5.4	5.0	51
**Cations’ ratios**							
	Ln	Co	Cr	Fe	Mn	Ni	O
La(Co,Cr,Fe,Mn,Ni)O_3−__δ_	-	0.191	0.249	0.199	0.178	0.182	-
Pr(Co,Cr,Fe,Mn,Ni)O_3−__δ_	-	0.196	0.191	0.200	0.209	0.204	-
Nd(Co,Cr,Fe,Mn,Ni)O_3−__δ_	-	0.184	0.204	0.216	0.208	0.188	-
Sm(Co,Cr,Fe,Mn,Ni)O_3−__δ_	-	0.202	0.198	0.221	0.206	0.174	-
Gd(Co,Cr,Fe,Mn,Ni)O_3−__δ_	-	0.219	0.185	0.196	0.208	0.192	-

**Table 3:** Thermal expansion coefficient of Ln(Co,Cr,Fe,Mn,Ni)O_3−δ_ series (heating run) evaluated in 200–900 °C range.

Composition	TEC (10^−6^ K^−1^) ((Heating) Run)	TEC (10^−6^ K^−1^) ((Cooling) Run)
La(Co,Cr,Fe,Mn,Ni)O_3−δ_	15.4(1)	15.4(1)
Pr(Co,Cr,Fe,Mn,Ni)O_3−δ_	14.6(1)	14.4(1)
Nd(Co,Cr,Fe,Mn,Ni)O_3−δ_	14.8(1)	14.7(1)
Sm(Co,Cr,Fe,Mn,Ni)O_3−δ_	14.6(1)	14.4(1)
Gd(Co,Cr,Fe,Mn,Ni)O_3−δ_	14.1(1)	14.0(1)

**Table 4:** The summary of the electrical conductivity properties of the Ln(Co,Cr,Fe,Mn,Ni)O_3−δ_ series, including the maximum measured value of conductivity *σ_max_*. The results for exemplary, conventional LnTMO_3_ perovskites are provided for comparison.

Composition	*E_a_* (eV)	*T* (°C)	*σ_max_* (S·cm^−1^)
La(Co,Cr,Fe,Mn,Ni)O_3−__δ_	0.363(3)	150–900	3.2
Pr(Co,Cr,Fe,Mn,Ni)O_3−__δ_	0.362(3)	150–700	2.3
Nd(Co,Cr,Fe,Mn,Ni)O_3−__δ_	0.365(3)	150–700	1.6
Sm(Co,Cr,Fe,Mn,Ni)O_3−__δ_	0.349(2)	200–900	1.2
Gd(Co,Cr,Fe,Mn,Ni)O_3−__δ_	0.417(2)	250–900	1.5

## Supplementary Materials

The following are available online at https://www.mdpi.com/article/10.3390/ma14185264/s1. Figure S1. Results of the TG measurement for Ln(Co,Cr,Fe,Mn,Ni)O_3−δ_ materials; Figure S2. The average TM-O distance vs the maximum value of electrical conductivity for Ln(Co,Cr,Fe,Mn,Ni)O3-δ series. The spreads resulting from the presence of multiple types of TM-O bonds in the distorted perovskite structures are also presented; Table S1. The results of the Rietveld refinement with the procedure’s residuals for the Ln_1−x_Sr_x_ (Co,Cr,Fe,Mn,Ni)O_3−δ_ (Ln = La, Pr, Nd, Sm, Gd) series-as-calcined powders; Table S2. The results of the Rietveld refinement with the procedure’s residuals for the Ln_1−x_Sr_x_(Co,Cr,Fe,Mn,Ni)O_3−δ_ (Ln = La, Pr, Nd, Sm, Gd) series-pellets quenched after sintering at 1000 °C for 20 h; Figure S3. XRD diffractograms of the Pr_1−*x*_Sr*_x_*(Co,Cr,Fe,Mn,Ni)O_3−δ_ series, for both as-calcined powders and pellets, sintered at 1000 °C for 20 h and quenched to RT; Figure S4. XRD diffractograms of the Nd_1−*x*_Sr*_x_*(Co,Cr,Fe,Mn,Ni)O_3−δ_ series, for both as-calcined powders and pellets, sintered at 1000 °C for 20 h and quenched to RT; Figure S5. XRD diffractograms of the Sm_1−*x*_Sr*_x_*(Co,Cr,Fe,Mn,Ni)O_3−δ_ series, for both as-calcined powders and pellets, sintered at 1000 °C for 20 h and quenched to RT; Figure S6. XRD diffractograms of the Gd_1−*x*_Sr*_x_*(Co,Cr,Fe,Mn,Ni)O_3−δ_ series, for both as-calcined powders and pellets, sintered at 1000 °C for 20 h and quenched to RT; Figure S7. Results of the EDS mappings for Pr_0.8_Sr_0.2_(Co,Cr,Fe,Mn,Ni)O_3−δ_; pellet sintered at 1000 °C for 20 h followed by quenching. The formation of secondary phase is clearly visible.
