# Supplementary material for: Formation of Solid Solutions and Physicochemical Properties of the High-Entropy Ln1−xSrx(Co,Cr,Fe,Mn,Ni)O3−δ (Ln = La, Pr, Nd, Sm or Gd) Perovskites
Source: Materials (Basel). 2021 Sep 13;14(18):5264. doi: 10.3390/ma14185264 (PMC8470994; doi:10.3390/ma14185264)
Supplement: Supplementary file 1 [file materials-14-05264-s001.zip › materials-1369836-supplementary.pdf]

materials

MDPI

Supplementary Material

Formation of Solid Solutions and Physicochemical Properties of the High-Entropy $Ln_{1-x}Sr_x(Co,Cr,Fe,Mn,Ni)O_{3-\delta}$ (Ln = La, Pr, Nd, Sm or Gd) Perovskites

Juliusz Dąbrowa [1,*], Klaudia Zielińska [2,*], Anna Stępień [2], Marek Zajusz [1], Margarita Nowakowska [1], Maciej Moździerz [2], Katarzyna Berent [3], Maria Szymczak [1] and Konrad Świerczek [2,4]

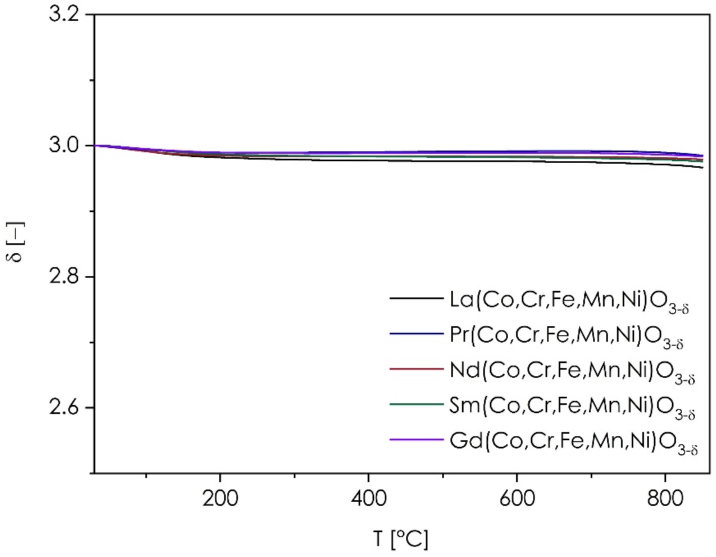

Figure S1. Results of the TG measurement for $Ln(Co,Cr,Fe,Mn,Ni)O_{3-\delta}$ materials.

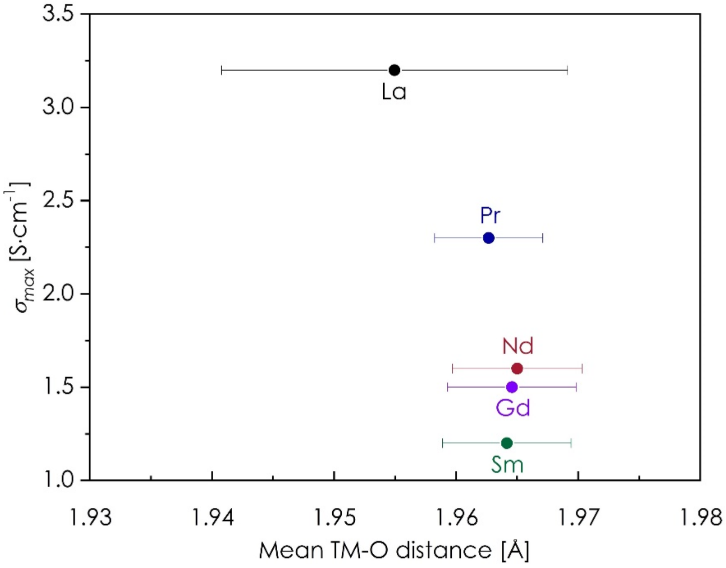

Figure S2. The average TM-O distance vs the maximum value of electrical conductivity for Ln(Co,Cr,Fe,Mn,Ni)O3-δ series. The spreads resulting from the presence of multiple types of TM-O bonds in the distorted perovskite structures are also presented.

Table S1. The results of the Rietveld refinement with the procedure's residuals for the $Ln_{1-x}Sr_x$ (Co,Cr,Fe,Mn,Ni)$O_{3-\delta}$ (Ln = La, Pr, Nd, Sm, Gd) series – as-calcined powders.

	Phase	Wt%	*a* [Å]	*b* [Å]	*c* [Å]	*V* [Å^3]	a_0 [Å]	Rwp [%]	Gof
**La-based series [1]**									
La(Co,Cr,Fe,Mn,Ni)O_3	*R*-3*c*	100	5.5086(2)		13.371(1)	58.563(6)	3.8834(1)	2.82	1.05
$La_{0.9}Sr_{0.1}$(Co,Cr,Fe,Mn,Ni)O_3	*R*-3*c*	100	5.5052(3)		13.363(1)	58.454(8)	3.8809(1)	2.88	1.20
$La_{0.8}Sr_{0.2}$(Co,Cr,Fe,Mn,Ni)O_3	*R*-3*c*	100	5.4997(3)		13.346(1)	58.27(1)	3.8768(1)	2.74	1.18
$La_{0.7}Sr_{0.3}$(Co,Cr,Fe,Mn,Ni)O_3	*R*-3*c*	100	5.5063(3)		13.362(2)	58.47(1)	3.8814(1)	2.86	0.96
$La_{0.6}Sr_{0.4}$(Co,Cr,Fe,Mn,Ni)O_3	*R*-3*c*	-	5.5067(6)		13.372(3)	58.53(2)	3.8826(1)	2.82	1.50
$La_{0.5}Sr_{0.5}$(Co,Cr,Fe,Mn,Ni)O_3	*R*-3*c*	-	5.5049(7)		13.380(4)	58.53(2)	3.8826(1)	2.97	1.82
**Pr-based series**									
	Phase	Wt%	*a* [Å]	*b* [Å]	*c* [Å]	*V* [Å^3]	a_0 [Å]	Rwp	Gof
Pr(Co,Cr,Fe,Mn,Ni)O_3	*Pbnm*	100	7.6952(3)	5.4713(2)	5.4405(2)	57.264(4)	3.8544(1)	3.11	3.18
$Pr_{0.9}Sr_{0.1}$(Co,Cr,Fe,Mn,Ni)O_3	*Pbnm*	98.5	76936(4)	5.4647(3)	5.4410(3)	57.185(5)	3.8527(1)	3.09	3.62
	*P*21/*n*	1.5	7.08(1)	7.69(2)	6.6993(3)				
$Pr_{0.8}Sr_{0.2}$(Co,Cr,Fe,Mn,Ni)O_3	*Pbnm*	96.0	7.6979(8)	5.4474(6)	5.4382(5)	57.011(9)	3.8488(2)	3.54	5.35
	*P*21/*n*	4.0	7.1165(6)	7.6624(8)	6.8033(5)				
**Nd-based series**									
	Phase	Wt%	*a* [Å]	*b* [Å]	*c* [Å]	*V* [Å^3]	a_0 [Å]	Rwp	Gof
Nd(Co,Cr,Fe,Mn,Ni)O_3	*Pbnm*	100	7.6731(3)	5.4781(2)	5.4100(2)	56.850(3)	3.8435(1)	3.29	1.49
$Nd_{0.9}Sr_{0.1}$(Co,Cr,Fe,Mn,Ni)O_3	*Pbnm*	100	7.6636(3)	5.4627(2)	5.4114(3)	56.636(5)	3.8403(1)	4.63	3.12
$Nd_{0.8}Sr_{0.2}$(Co,Cr,Fe,Mn,Ni)O_3	*Pbnm*	94.3	7.6638(5)	5.4556(3)	5.4108(3)	56.557(6)	3.8385(1)	4.33	3.12
	P21/n	5.7	7.0636(8)	7.5516(2)	6.7160(1)				
**Sm-based series**									
	Phase	Wt%	*a* [Å]	*b* [Å]	*c* [Å]	*V* [Å^3]	a_0 [Å]	Rwp	Gof
Sm(Co,Cr,Fe,Mn,Ni)O_3	*Pbnm*	100	7.6171(4)	5.5061(3)	5.3523(2)	56.120(4)	3.8285(1)	2.57	1.23
$Sm_{0.9}Sr_{0.1}$(Co,Cr,Fe,Mn,Ni)O_3	*Pbnm*	97.2	7.6159(3)	5.5040(2)	5.3530(2)	56.097(4)	3.8281(1)	2.53	1.25
	*P*21/*n*	2.7	7.3306(2)	7.0044(2)	5.5982(2)				
**Gd-based series**									
	Phase	Wt%	*a* [Å]	*b* [Å]	*c* [Å]	*V* [Å^3]	a_0 [Å]	Rwp	Gof
Gd(Co,Cr,Fe,Mn,Ni)O_3	*Pbnm*	100	7.5725(4)	5.5371(3)	5.2967(2)	55.526(4)	3.8150(1)	2.08	1.21
$Gd_{0.9}Sr_{0.1}$(Co,Cr,Fe,Mn,Ni)O_3	*Pbnm*	100	7.5690(4)	5.5360(3)	5.5293(3)	55.481(5)	3.8140(1)	2.22	1.40
$Gd_{0.8}Sr_{0.2}$(Co,Cr,Fe,Mn,Ni)O_3	*Pbnm*	98.3	7.571(4)	5.5288(3)	5.3016(3)	55.478(5)	3.3139(1)	1.89	1.09
	*P*21/*n*	1.7	7.3213(3)	6.9956(3)	5.5927(2)				

Table S2. The results of the Rietveld refinement with the procedure's residuals for the $Ln_{1-x}Sr_x(Co,Cr,Fe,Mn,Ni)O_{3-\delta}$ (Ln = La, Pr, Nd, Sm, Gd) series – pellets quenched after sintering at 1000 °C for 20 h.

	Phase	Wt%	*a* [Å]	*b* [Å]	*c* [Å]	*V* [Å³]	*a₀* [Å]	Rwp [%]	Gof
**La-based series [1]**									
La(Co.Cr.Fe.Mn.Ni)O_3	*Pbnm*	100	7.7472(2)	5.5116(1)	5.4671(1)	58.360(2)	3.8789(1)	7.17	2.01
$La_{0.9}Sr_{0.1}$(Co.Cr.Fe.Mn.Ni)O_3	*R-3c*	100	5.4966(1)		13.2676(2)	57.858(2)	3.8677(1)	8.38	2.96
$La_{0.8}Sr_{0.2}$(Co.Cr.Fe.Mn.Ni)O_3	*R-3c*	100	5.4845(1)		13.2785(4)	57.651(3)	3.8631(1)	7.38	2.33
$La_{0.7}Sr_{0.3}$(Co.Cr.Fe.Mn.Ni)O_3	*R-3c*	100	5.4738(1)		13.2814(4)	57.480(3)	3.8693(1)	6.95	2.3
$La_{0.6}Sr_{0.4}$(Co.Cr.Fe.Mn.Ni)O_3	*R-3c*	94.2	5.4688(2)		13.3014(5)	57.420(4)	3.8579(1)	6.08	1.62
	R-3m	4.6	5.542(2)		20.252(3)				
	Fm-3m	1.2	4.1825(5)						
$La_{0.5}Sr_{0.5}$(Co.Cr.Fe.Mn.Ni)O_3	*R-3c*	87.1	5.4648(2)		13.3224(8)	57.427(5)	3.8581(1)	6.23	1.98
	R-3m	10.6	5.586(6)		20.238(4)				
	Fm-3m	2.3	4.1826(2)						
**Pr-based series**									
	Phase	Wt%	*a* [Å]	*b* [Å]	*c* [Å]	*V* [Å³]	*a₀* [Å]	Rwp	Gof
Pr(Co.Cr.Fe.Mn.Ni)O_3	*Pbnm*	100	7.6976(1)	5.4673(1)	5.4413(1)	57.249(1)	3.8541(1)	3.44	4.31
$Pr_{0.9}Sr_{0.1}$(Co.Cr.Fe.Mn.Ni)O_3	*Pbnm*	100	7.6889(1)	5.4416(1)	5.4463(1)	56.969(7)	3.8478(1)	3.39	4.94
$Pr_{0.8}Sr_{0.2}$(Co.Cr.Fe.Mn.Ni)O_3	*Pbnm*	96.2	7.6833(1)	5.4375(1)	5.4456(1)	56.876(2)	3.8457(4)	3.21	4.43
	P21/n	3.8	7.118(8)	7.739(7)	6.701(1)				
**Nd-based series**									
	Phase	Wt%	*a* [Å]	*b* [Å]	*c* [Å]	*V* [Å³]	*a₀* [Å]	Rwp	Gof
Nd(Co.Cr.Fe.Mn.Ni)O_3	*Pbnm*	100	7.6727(1)	5.4751(1)	5.4098(1)	56.814(1)	3.8443(1)	3.87	2.37
$Nd_{0.9}Sr_{0.1}$(Co.Cr.Fe.Mn.Ni)O_3	*Pbnm*	100	7.6691(1)	5.4525(1)	5.4178(1)	56.637(2)	3.8403(1)	4.04	2.77
$Nd_{0.8}Sr_{0.2}$(Co.Cr.Fe.Mn.Ni)O_3	*Pbnm*	95.8	7.6673(3)	5.4473(2)	5.4183(2)	56.577(4)	3.8389(1)	4.78	4.03
	P21/n	4.2	7.13(2)	7.77(1)	6.7015(8)				
**Sm-based series**									
	Phase	Wt%	*a* [Å]	*b* [Å]	*c* [Å]	*V* [Å³]	*a₀* [Å]	Rwp	Gof
Sm(Co.Cr.Fe.Mn.Ni)O_3	*Pbnm*	100	7.6178(1)	5.4986(1)	5.3533(1)	56.060(1)	3.8272(1)	3.02	1.93
$Sm_{0.9}Sr_{0.1}$(Co.Cr.Fe.Mn.Ni)O_3	*Pbnm*	98.2	7.6263(3)	5.4776(2)	5.3669(2)	56.048(4)	3.8270(1)	3.74	3.04
	P21/n	1.8	7.3391(6)	7.0064(6)	5.5998(3)				
**Gd-based series**									
	Phase	Wt%	*a* [Å]	*b* [Å]	*c* [Å]	*V* [Å³]	*a₀* [Å]	Rwp	Gof
Gd(Co.Cr.Fe.Mn.Ni)O_3	*Pbnm*	100	7.5726(1)	5.5293(1)	5.2980(1)	55.458(1)	3.8135(1)	2.45	1.78
$Gd_{0.9}Sr_{0.1}$(Co.Cr.Fe.Mn.Ni)O_3	*Pbnm*	100	7.5782(3)	5.5111(2)	5.3093(2)	55.435(4)	3.3130(1)	2.79	2.31
$Gd_{0.8}Sr_{0.2}$(Co.Cr.Fe.Mn.Ni)O_3	*Pbnm*	97.7	7.5836(3)	5.5048(2)	5.3161(2)	55.481(4)	3.8140(1)	2.43	1.73
	P21/n	2.3	7.1227(8)	7.2674(9)	6.9344(3)				

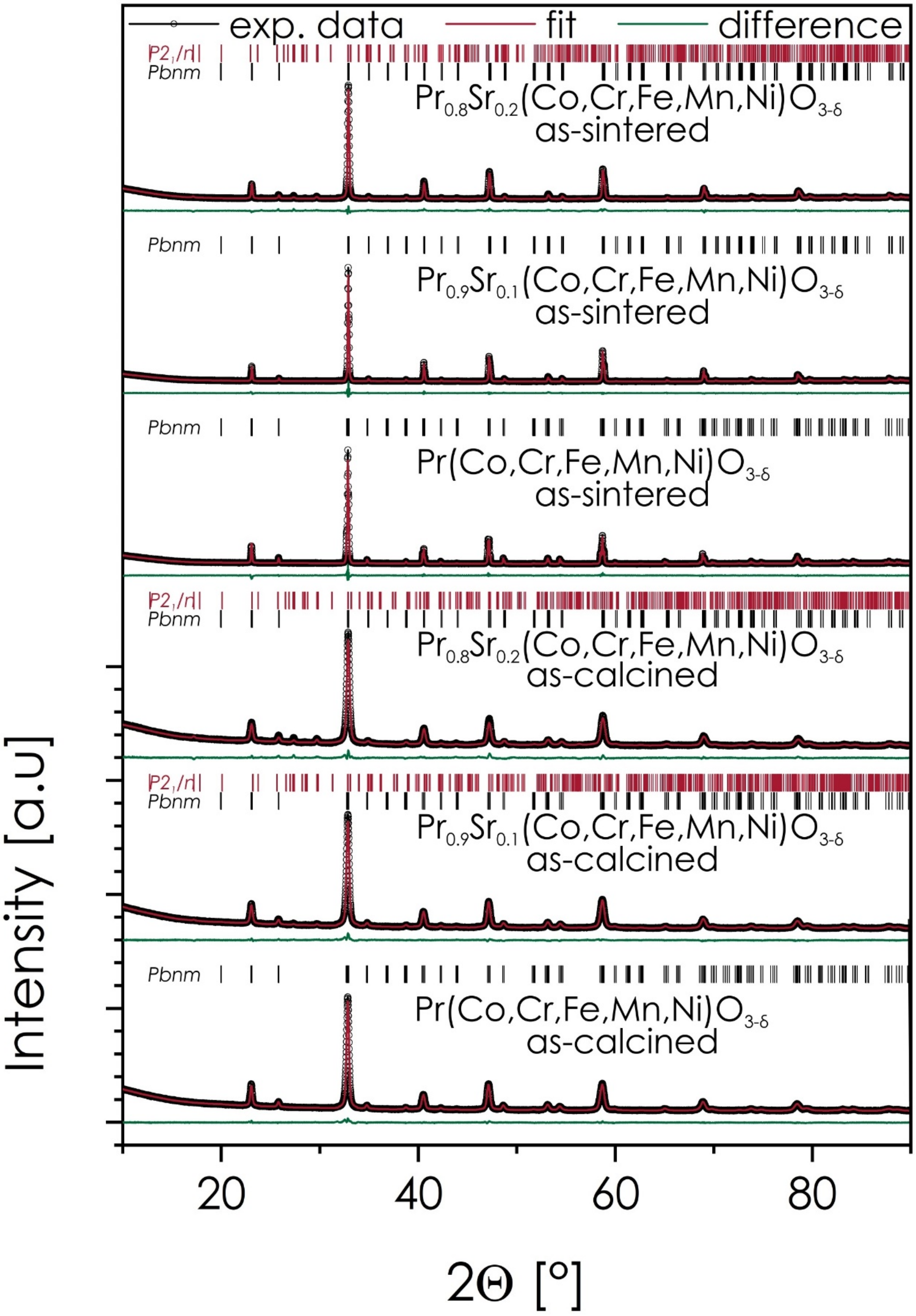

Figure S3. XRD diffractograms of the $Pr_{1-x}Sr_x(Co,Cr,Fe,Mn,Ni)O_{3-\delta}$ series, for both as-calcined powders and pellets, sintered at 1000 °C for 20 h and quenched to RT.

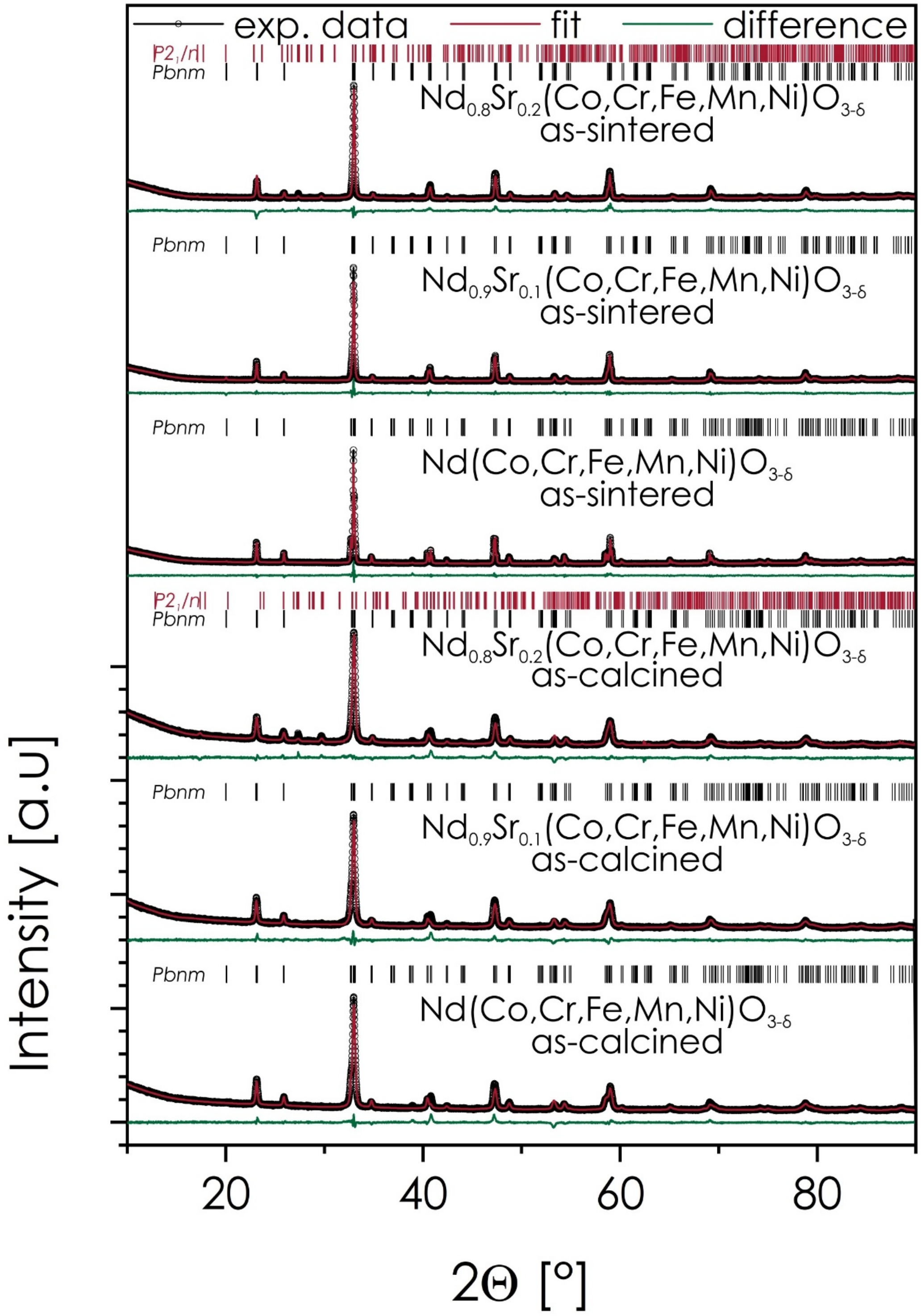

Figure S4. XRD diffractograms of the $Nd_{1-x}Sr_x(Co,Cr,Fe,Mn,Ni)O_{3-δ}$ series, for both as-calcined powders and pellets, sintered at 1000 °C for 20 h and quenched to RT.

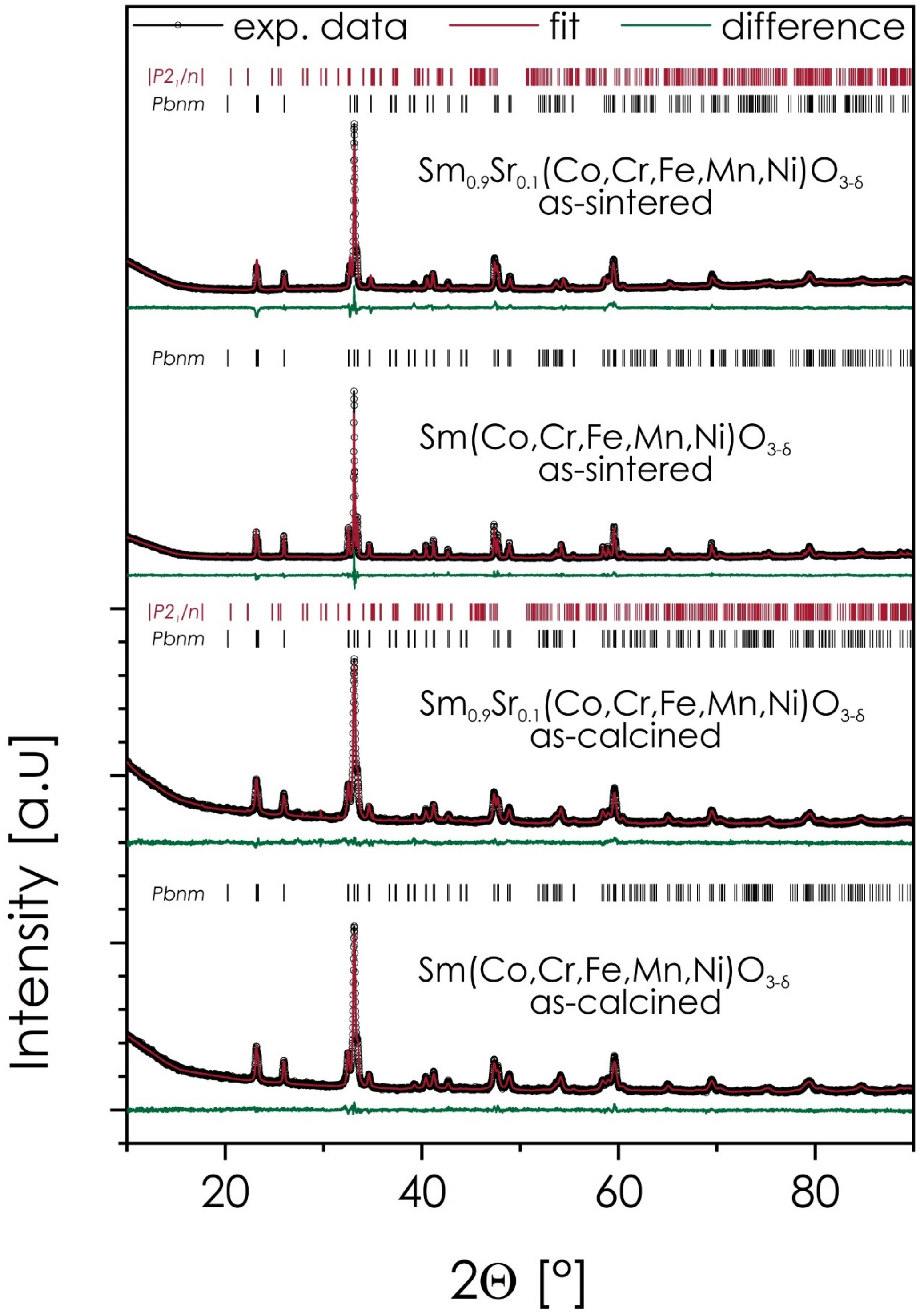

Figure S5. XRD diffractograms of the $Sm_{1-x}Sr_x(Co,Cr,Fe,Mn,Ni)O_{3-\delta}$ series, for both as-calcined powders and pellets, sintered at 1000 °C for 20 h and quenched to RT.

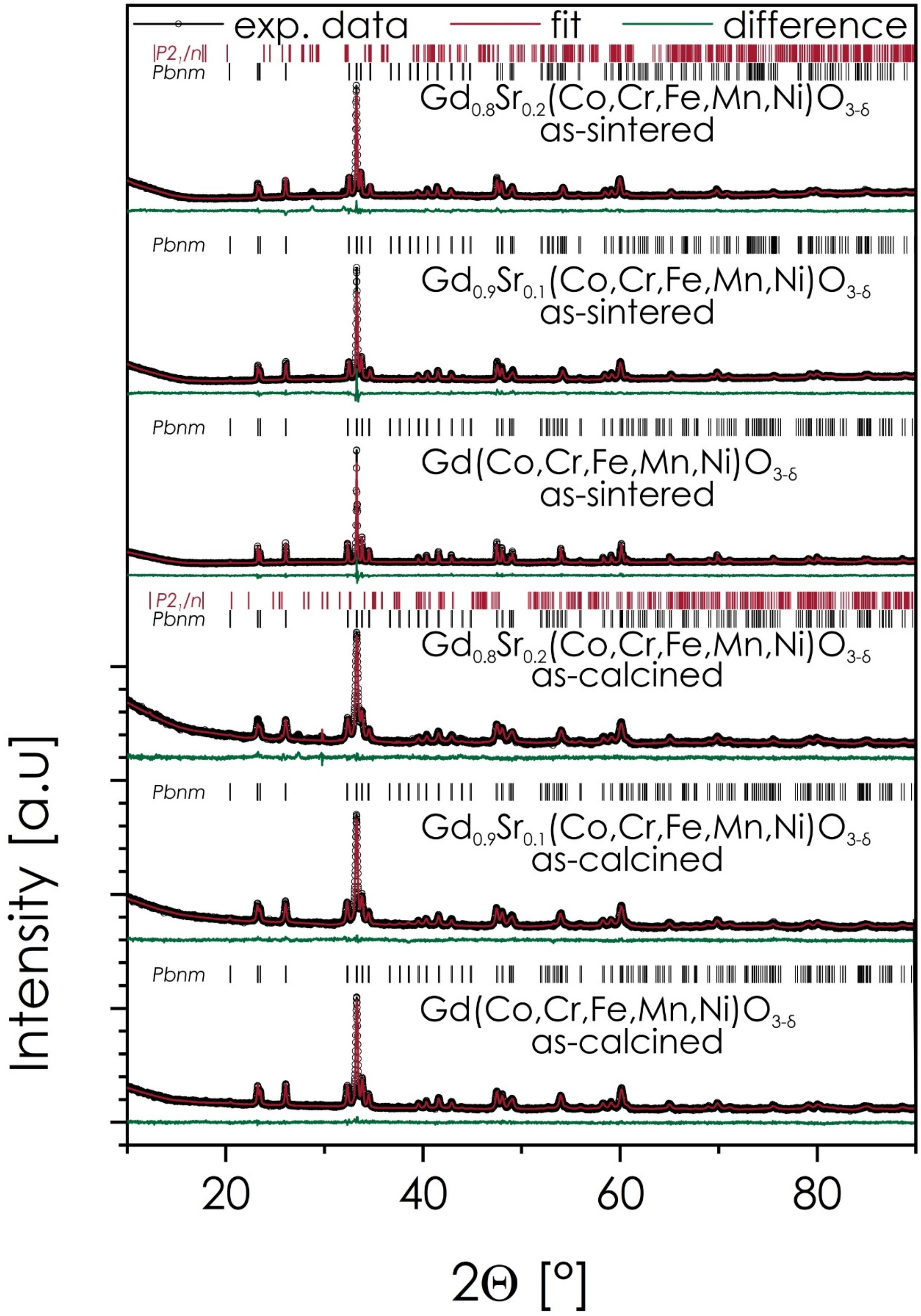

Figure S6. XRD diffractograms of the $Gd_{1-x}Sr_x(Co,Cr,Fe,Mn,Ni)O_{3-\delta}$ series, for both as-calcined powders and pellets, sintered at 1000 °C for 20 h and quenched to RT.

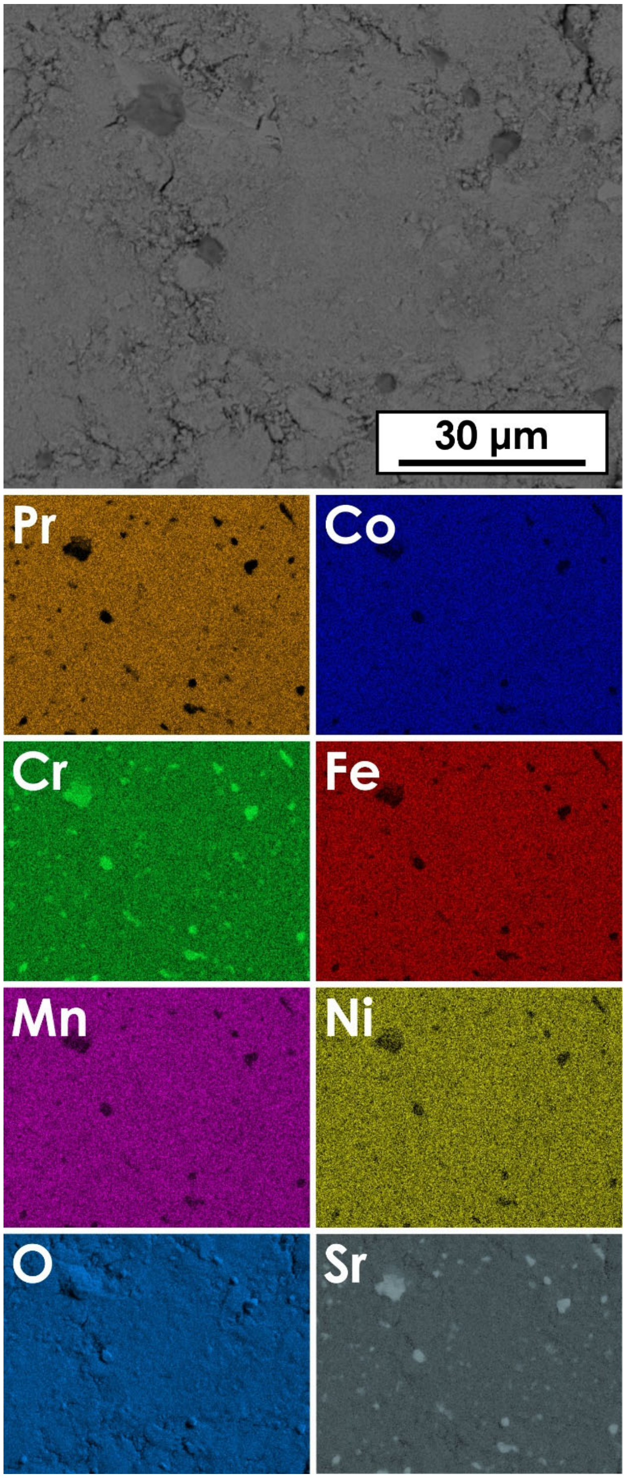

Figure S7. Results of the EDS mappings for $Pr_{0.8}Sr_{0.2}(Co,Cr,Fe,Mn,Ni)O_{3-\delta}$; pellet sintered at 1000 °C for 20 h followed by quenching. The formation of secondary phase is clearly visible.
